# Early and Long-Term HIV-1 Immunogenicity Induced in Macaques by the Combined Administration of DNA, NYVAC and Env Protein-Based Vaccine Candidates: The AUP512 Study

**DOI:** 10.3389/fimmu.2022.939627

**Published:** 2022-07-22

**Authors:** Beatriz Perdiguero, Benedikt Asbach, Carmen E. Gómez, Josef Köstler, Susan W. Barnett, Marguerite Koutsoukos, Deborah E. Weiss, Anthony D. Cristillo, Kathryn E. Foulds, Mario Roederer, David C. Montefiori, Nicole L. Yates, Guido Ferrari, Xiaoying Shen, Sheetal Sawant, Georgia D. Tomaras, Alicia Sato, William J. Fulp, Raphael Gottardo, Song Ding, Jonathan L. Heeney, Giuseppe Pantaleo, Mariano Esteban, Ralf Wagner

**Affiliations:** ^1^ Department of Molecular and Cellular Biology, Centro Nacional de Biotecnología, Consejo Superior de Investigaciones Científicas, Madrid, Spain; ^2^ Centro de Investigación Biomédica en Red de Enfermedades Infecciosas, Instituto de Salud Carlos III (CIBERINFEC, ISCIII ), Madrid, Spain; ^3^ Institute of Medical Microbiology and Hygiene, University of Regensburg, Regensburg, Germany; ^4^ Institute of Clinical Microbiology and Hygiene, University Hospital Regensburg, Regensburg, Germany; ^5^ Novartis Vaccines, Cambridge, MA, United States; ^6^ Department of Product Development, GlaxoSmithKline (GSK) Vaccines, Rixensart, Belgium; ^7^ Department of Immunobiology, Advanced BioScience Laboratories (ABL) Inc., Rockville, MD, United States; ^8^ Vaccine Research Center, National Institute of Allergy and Infectious Diseases, National Institutes of Health, Bethesda, MD, United States; ^9^ Duke Human Vaccine Institute and Department of Surgery, Duke University Medical Center, Durham, NC, United States; ^10^ Vaccine and Infectious Disease Division, Fred Hutchinson Cancer Research Center, Seattle, WA, United States; ^11^ Biomedical Data Sciences, Lausanne University Hospital, University of Lausanne, Lausanne, Switzerland; ^12^ Translational Data Science, Swiss Institute of Bioinformatics, Lausanne, Switzerland; ^13^ EuroVacc Foundation EuroVacc Programme Coordinator, Lausanne, Switzerland; ^14^ Lab of Viral Zoonotics, Department of Veterinary Medicine, University of Cambridge, Cambridge, United Kingdom; ^15^ Division of Immunology and Allergy, Department of Medicine, Centre Hospitalier Universitaire Vaudois, University of Lausanne, Lausanne, Switzerland

**Keywords:** HIV-1 vaccine, DNA and NYVAC vectors, recombinant gp120 protein, combined immunization regimens, immunogenicity, B and T cell responses, non-human primates

## Abstract

To control HIV infection there is a need for vaccines to induce broad, potent and long-term B and T cell immune responses. With the objective to accelerate and maintain the induction of substantial levels of HIV-1 Env-specific antibodies and, at the same time, to enhance balanced CD4 and CD8 T cell responses, we evaluated the effect of concurrent administration of MF59-adjuvanted Env protein together with DNA or NYVAC vectors at priming to establish if early administration of Env leads to early induction of antibody responses. The primary goal was to assess the immunogenicity endpoint at week 26. Secondary endpoints were (i) to determine the quality of responses with regard to RV144 correlates of protection and (ii) to explore a potential impact of two late boosts. In this study, five different prime/boost vaccination regimens were tested in rhesus macaques. Animals received priming immunizations with either NYVAC or DNA alone or in combination with Env protein, followed by NYVAC + protein or DNA + protein boosts. All regimens induced broad, polyfunctional and well-balanced CD4 and CD8 T cell responses, with DNA-primed regimens eliciting higher response rates and magnitudes than NYVAC-primed regimens. Very high plasma binding IgG titers including V1/V2 specific antibodies, modest antibody-dependent cellular cytotoxicity (ADCC) and moderate neutralization activity were observed. Of note, early administration of the MF59-adjuvanted Env protein in parallel with DNA priming leads to more rapid elicitation of humoral responses, without negatively affecting the cellular responses, while responses were rapidly boosted after repeated immunizations, indicating the induction of a robust memory response. In conclusion, our findings support the use of the Env protein component during priming in the context of an heterologous immunization regimen with a DNA and/or NYVAC vector as an optimized immunization protocol against HIV infection.

## 1 Introduction

Despite major efforts in the pursuit of an HIV-1 vaccine, there is no such modality with sufficient efficacy yet. The RV144 phase III clinical trial in Thailand is the only one that showed modest efficacy of 31.2% at 3.5 years after vaccination. This prophylactic trial used a combination of immunogens, the canarypoxvirus vector ALVAC during prime and boost, and alum-adjuvanted monomeric gp120 Env protein (AIDSVAX B/E) during boost (1). Correlates of protection identified in the trial were IgG binding antibodies to Env targeting the V1/V2 loop, and antibodies mediating antibody-dependent cellular cytotoxicity (ADCC) when plasma IgA levels were low. This trial also provided evidence that protection from HIV-1 infection after one year was in the order of 60%, and thereafter waned with time, associated with a drop in binding antibodies to V1/V2 (1). Further studies with RV144 samples showed that IgG3 binding antibody responses to gp120 correlated with reduced risk of virus infection (2), as did polyfunctional CD4^+^ T cells (3). These findings highlight that a vaccine might be possible if more potent immunogens and optimized protocols of immunization are used. This prompted the development of additional immunogens based on viral and non-viral vectors expressing HIV-1 antigens (4–8).

Due to the high incidence of HIV-1 infections in South Africa, an effort has been put forward to perform efficacy trials in this country where clade C is prominent. A follow up phase 2b/3 clinical trial (HVTN 702) with ALVAC vector, but now boosted with MF59-adjuvanted bivalent gp120 of clade C, however, failed to show efficacy in an interim analysis and was stopped (9). Currently, it is unclear whether this failure is related to differences in the study population compared to RV144 or to other factors. Another phase 2b/3 trial to test vaccine efficacy in women (HVTN 705 study; https://www.clinicaltrials.gov/ct2/show/NCT03060629) has been stopped due to lack of efficacy, and the other trial in homosexual men (HVTN 706 study/MOSAICO; https://www.clinicaltrials.gov/ct2/show/NCT03964415) is underway. Vaccine candidates comprise an Adenovirus-26 vector expressing mosaic antigens for prime and boost, and trimeric gp140 Env protein components for boost (alum-adjuvanted C clade gp140 in HVTN 705, and bivalent clade C and mosaic gp140 in HVTN 706). Whether the use of Ad-26 vector and the modified immunogens provide any significant improvements in the control of HIV-1 infection rates remains to be established.

With the goal to accelerate, maintain and balance the induction of HIV-1-specific T and B cell responses, we have characterized the effect of combining three vaccine components (DNA, NYVAC and Env protein) encoding a corresponding set of HIV-1 clade C antigens in five different immunization regimens in non-human primates (NHPs). In previous HIV-1 vaccination studies in NHPs, the administration of DNA or NYVAC vectors expressing HIV antigens for priming, followed by a booster with the same vectors together with Env protein, have shown that priming with a DNA vector frames the quality of the immune responses prior to a poxvirus and protein boost (8, 10, 11). Thus, the main question addressed in this study is whether co-administration of MF59-adjuvanted Env protein already during the priming could accelerate the induction of potent antibody responses. Secondary endpoints were (i) to determine the quality of responses in particular with regard to RV144 correlates of protection and (ii) to explore a potential impact of two late boosts.

Our findings in NHPs provide insight into how different combinations of vectors, i. e. nucleic acid, a non-replicating attenuated virus vector and an MF59-adjuvanted Env protein component, affect the potency of B and T cell immune responses during prime/boost and follow up for 50 weeks. Moreover, our results support the incorporation of the protein component together with the DNA in the prime followed by NYVAC + protein in the boost as the most effective vaccination regimen to induce an early humoral immune response against HIV-1 immunogens.

## 2 Materials and methods

### 2.1 Animals and Ethic Statement

**Figure 1:**
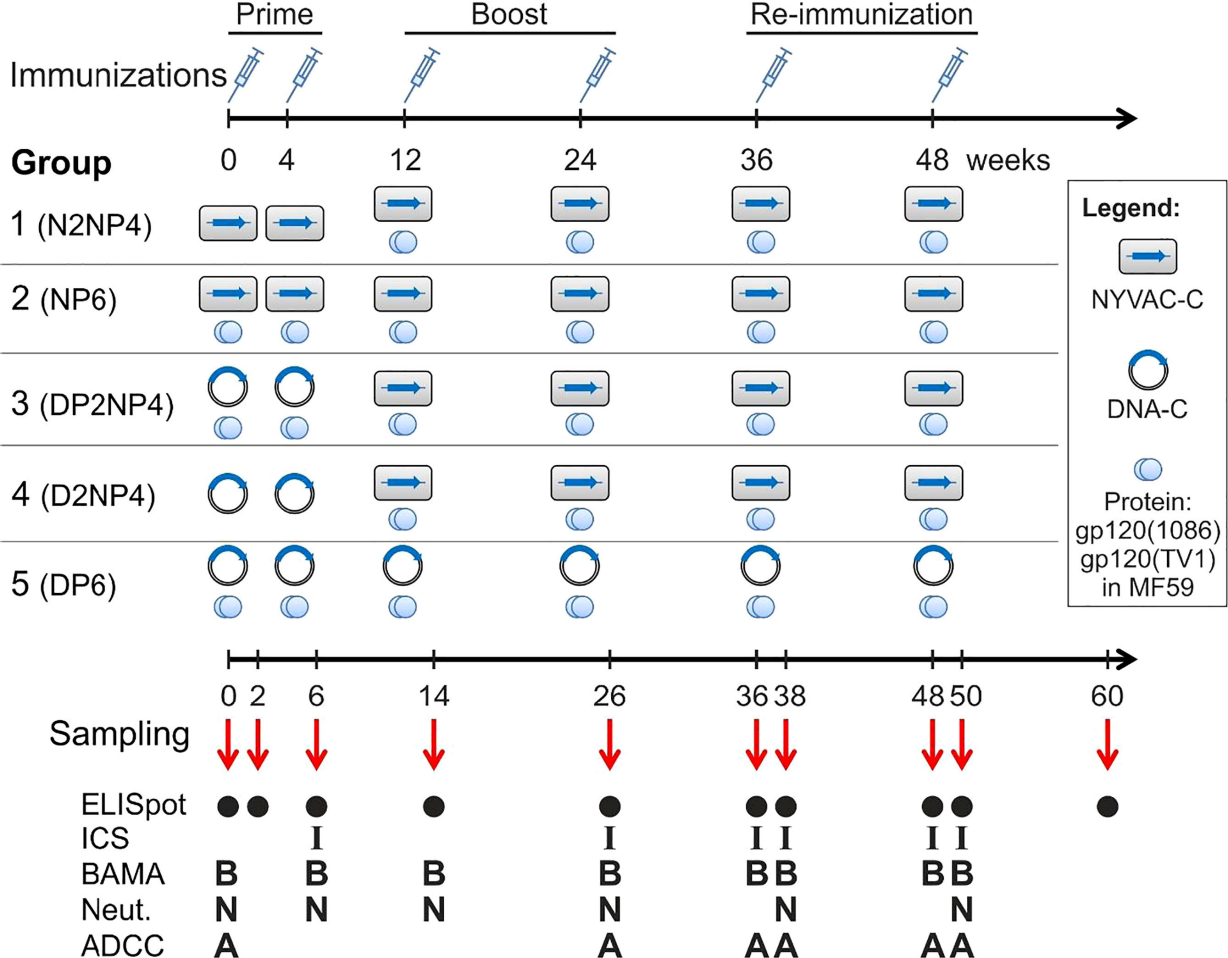
Study design, immunization groups and immunogenicity endpoints of the AUP512 NHP Study. Immunizations with different combinations of candidate HIV-1 vaccines constituting DNA, NYVAC and Env protein were performed by the intramuscular (i.m.) route at weeks 0, 4, 12, 24, 36 and 48. Blood and rectal mucosal secretions were collected for ELISpot, ICS or antibody analyses at the indicated time-points.

The potency of the vaccine candidates was assessed in male Indian rhesus macaques (*Macaca mulatta mulatta*) that were housed, fed, given environmental enrichment and handled at the Advanced Bioscience Laboratories’ Animal Facility (ABL, Inc.; Rockville, MD, USA). The age of the animals ranged between 2.5 and 2.9 years, with a mean of 2.6 years, and the weight range was between 3.1 to 5.7 kg, with a mean of 3.8 kg. Tests undertaken by ABL showed that all rhesus macaques were negative for tuberculosis, simian retrovirus (SRV), simian T-cell leukemia virus 1 (STLV-1), herpesvirus B, simian immunodeficiency virus (SIV) and measles, and also demonstrated negative fecal culture for *Salmonella*, *Shigella*, *Campylobacter* and *Yersinia* genera. Furthermore, animals were immunologically naive for the vaccine components. ABL, Inc.’s Institutional Animal Care and Use Committee approved the study under protocol number AUP512. The study used 52 rhesus macaques divided into 5 groups: 12 macaques each in groups 1, 2 and 3, and 8 macaques each in groups 4 and 5. The immunization schedule and immunization groups are detailed in **Figure 1**.

All procedures and primate colony care strictly adhered to the recommendations found in the 8^th^ edition of the Guide for the Care and Use of Laboratory Animals (National Research Council) (12), as well as in the Public Health Services Policy on the Humane Care and Use of Laboratory Animals from the Office of Animal Welfare (part of the USA Department of Health and Human Services), with all activities being in full compliance with the regulations found in the Animal Welfare Act (9 CFR 3.81).

Animals were given Telazol (4-6 mg/kg of body weight) or Ketamine (10 mg/kg) for routine procedures (physicals, injections, phlebotomies). Sedation was performed using Domitor (0.03 mg/kg), Acepromazine (0.5-1 mg/kg) or Propofol (to effect) with Antisedan (0.3 mg/kg) as a partial Domitor reversal agent and Isoflurane (to effect) inhalant anesthesia. Euthanasia overdose was given intravenously (i.v.) using barbiturate euthanasia agent. Anesthesia and euthanasia were given by trained personnel under the supervision of veterinary staff. Recommendations by the Weatherall report on the use of NHPs in research (13) were followed and extended by ABL’s Primate Environmental Enrichment Program to ameliorate animal welfare and to minimize suffering.

Safety monitoring included observation for general behaviour and clinical symptoms, body weight and temperature and local reactions at injection sites. General behaviour and clinical symptoms (including appetite) were monitored daily during the whole experiment by trained personnel. Body weight and temperature were measured before the start of the experiment and immediately after sedation for immunizations or sample collections. Cage side observation for local reactions at injection sites occurred at pre-dosing, 10 minutes, 30 minutes, 1 hour and 24 hours and then daily for one-week post-dosing. At selected time-points, a physical examination by a veterinarian was performed, and clinical chemistry and hematology parameters were measured one week after each dosing by the clinical laboratories at ABL.

### 2.2 Antigens

Gag, PolNef and gp140 sequences have been previously described (11). The DNA vaccine consisted of a mixture of three plasmids carrying either the *gag* gene (HIV-1 clade C isolate 96ZM651) supporting formation and release of virus-like particles, the *gp140* gene (HIV-1 clade C isolate 96ZM651) resulting in the release of Env trimers from transduced cells or an artificial fusion of modified *pol* and *nef* gene (HIV-1 clade C/B’ isolate 97CN54) in pVRC-8400 (11).

The NYVAC vaccine consisted of two recombinant viruses, one with GagPolNef [NYVAC-Gag(96ZM651)-Pol-Nef(97CN54)] and the other with gp140 [NYVAC-gp140(96ZM651)] HIV-1 genes inserted into the thymidine kinase (TK) locus (*J2R* gene) of New York Vaccinia Virus [NYVAC; (14)] as previously described (15). Viruses were produced in baby hamster kidney (BHK)-21 cells and purified through two 36% (w/v) sucrose cushions. Titers were determined by immunostaining plaque assay in African green monkey BSC-40 cells, as previously described (16).

The protein vaccine consisted of a mix of two recombinant HIV-1 clade C gp120 proteins, derived from the TV1.C (chronic) and 1086.C (transmitted founder) virus isolates (17) expressed from stably transfected CHO cell lines, purified and characterized as previously described (18).

To facilitate the nomenclature along the study, we refer to the DNA vaccine as the mixture of three plasmids, the NYVAC vaccine as the mixture of two recombinant viruses and the protein vaccine as the mixture of two proteins.

### 2.3 Vaccine Administration

The trivalent DNA vaccine (provided by Prof. Dr. Ralf Wagner, University of Regensburg, Regensburg, Germany) was administered by intramuscular (i.m.) injection of 1 mL in the upper left and 1 mL in the upper right legs (2 mg/mL in phosphate-buffered saline -PBS-).

The bivalent poxvirus NYVAC vaccine (provided by Prof. Dr. Mariano Esteban, Centro Nacional de Biotecnología, Madrid, Spain) was administered by i.m. injection of 1 mL of a 2×10^8^ pfu/mL solution into the deltoid of the right arm of each animal.

The gp120 protein vaccine (provided by Novartis, Basel, Switzerland) in 0.5 mL containing 50 μg TV1.C gp120 + 50 μg 1086.C gp120 was mixed with an equal volume of MF59 adjuvant prior to administration. One mL of the MF59-adjuvanted protein mix was injected i.m. into the deltoid of the left arm of each animal (opposite to the NYVAC vaccine).

Vaccines were administered into different sites to allow independent assessment of local reactogenicity to the different agents as part of the safety monitoring analysis and to expand the local immune reactivity.

Blood samples for the different immunological analyses were obtained at the time-points indicated in **Figure 1**. For T cell analyses, EDTA-blood was collected, and for antibody determinations, plasma and clotted blood were harvested. Rectal mucosal secretions were collected *via* Weck-Cel sponges at the different time-points indicated in **Figure 1** at the same time of the collection of plasma/serum samples for the determination of Ig classes, particularly IgG and IgA.

### 2.4 Immunological Analyses

#### 2.4.1 Peptides

In accordance with previous studies, overlapping peptides (15-mers overlapping by 11 amino acids) spanning the Env, Gag, Pol and Nef HIV-1 clade C regions were matched to the inserts expressed by the different vaccine components and used in the IFN-γ ELISpot and ICS assays for T cell stimulation. Peptides were grouped into nine different peptide pools: Env-1 (96ZM651 Env peptides 1 to 55), Env-2 (peptides 56 to 110), Env-3 (peptides 111 to 166), Gag-1 (96ZM651 Gag peptides 1 to 68), Gag2/Pol (Gag peptides 69 to 121 and Pol 1 to 14 covering trans-frame within Gag, i.e. p6* and beginning of Pro of 96ZM651), GagPol (peptides 1 to 60 of Pol of 97CN54 with leading 10 aa of 97CN54 gag), Pol-1 (peptides 61 to 121), Pol-2 (peptides 122 to 183) and Nef (97CN54 Nef peptides 1 to 49) (8, 10, 11). They were provided by Centre Hospitalier Universitaire Vaudois (CHUV, Lausanne, Switzerland).

#### 2.4.2 IFN-γ Enzyme-Linked Immunosorbent Spot (ELISpot) Assay

The quantification of the HIV-1-specific IFN-γ-secreting cells at the different time-points indicated in **Figure 1** was performed at ABL, Inc. by ELISpot assay from freshly isolated peripheral blood mononuclear cells (PBMCs) stimulated *ex vivo* in triplicates with the different HIV-1 peptide pools (1 μg/mL). Negative control stimulation was medium alone and positive control stimulation used phytohaemagglutinin P (PHA; 2.5 µg/mL). Briefly, Millipore 96-well filtration plates were pre-treated with 70% EtOH, washed with PBS, coated with capture antibody (mouse anti-human IFN-γ, BD Pharmingen) at a concentration of 5 μg/mL and incubated overnight at 4°C. The next day, plates were blocked with complete Roswell Park Memorial Institute (RPMI) medium for at least 2 hours at 37°C, then washed with complete RPMI medium and HIV-1 peptide pools, PHA or medium alone together with PBMCs (2×10^5^ cells/well) were added to the appropriate wells. Plates were incubated at 37°C for 18-24 hours, washed twice with cold dH_2_0 and then five times with wash buffer (PBS with 0.05% Tween 20). Biotinylated anti-IFN-γ antibody (Mabtech) was added at a concentration of 1 μg/mL. Plates were incubated for 1 h at 37°C and then washed again with wash buffer. Avidin-horseradish peroxidase (HRP) (Vector Laboratories) was added at a 1:2,000 dilution and plates were again incubated for 1 h at 37°C. After a final wash, stable diaminobenzidine (DAB; Invitrogen) was added to the plates and spot development occurred for two minutes. Plates were finally washed thoroughly under tap water to stop the reaction and allowed to dry in the dark. The number of spots in each well was counted with an automated ELISpot reader (CTL ImmunoSpot v5 reader). Animals were classified as responders if more than 50 spot-forming units (SFUs) per million PBMCs and at least 4 times the week 0 background values for total HIV-1 response (sum of the different HIV-1 peptide pools) were measured.

#### 2.4.3 CD4 and CD8 T Cell ICS Analysis

EDTA-blood samples were harvested and PBMCs were prepared, viably frozen, cryostored and shipped to Vaccine Immune Monitoring Center at the Vaccine Research Center (VIMC-VRC) for the determination of the response rate and magnitude of the HIV-1-specific CD4 and CD8 T cell responses by Intracellular Cytokine Staining (ICS) assay and flow cytometry at weeks 6, 26, 36, 38, 48 and 50 (**Figure 1**) as previously described (19). Briefly, cryopreserved PBMCs were thawed and rested overnight in medium. For stimulation, the different HIV-1 peptide pools were added at 2 μg/mL in the presence of 10 μg/mL brefeldin A to 1-3×10^6^ cells in 96-well plates. As a negative control, cells were stimulated with dimethyl sulfoxide (DMSO; peptide pool buffer). After stimulation for 6 h at 37°C, cells were stored at 4°C until staining. For this, cells were fixed, permeabilized and stained with fluorescence-labeled antibodies directed against CD3, CD4, CD8, interferon-γ (IFN-γ), interleukin-2 (IL-2) and tumor necrosis factor (TNF). Aqua LIVE/DEAD kit (Invitrogen) was used to exclude dead cells. Samples were acquired on an LSR II flow cytometer and analyzed using FlowJo version 9.3 (Treestar, Inc.; Ashland, OR, USA). Samples were excluded from the flow cytometric analysis if the cell viability was less than 35% or if the events (total cells) acquired were less than 35,000. Animals were classified as responders if they exhibited a significant response against at least one peptide pool as compared to the DMSO control stimulation.

#### 2.4.4 HIV-1-Specific Binding Antibodies

HIV-1-specific antibody levels towards a set of readout antigens were measured by the CA-VIMC (Tomaras’s lab) using the standardized binding antibody multiplex assay (BAMA) as described previously (2, 20, 21). Distinct sets of carboxylated xMAP microspheres (Luminex, TX, USA) were covalently coupled to the antigens listed below, mixed and incubated with macaque plasma samples collected at weeks -2, 6, 14, 26, 36, 38, 48 and 50. The following antigens were coupled to microspheres: gp120 1086, gp120 TV1, gp140 A1.con.env03 CF, gp140 B.con.env03 CF, gp140 C.con.env03 CF, gp140 Con S CFI, gp140 00MSA4076, gp140 JRFL, C.1086C_V1_V2 Tags and gp70_B.CaseA_V1_V2 (hereinafter abbreviated as 1086, TV1, conA1, conB, conC, conM, 00MSA, JRFL, 1086-V1V2 and CaseA-V1V2, respectively). The negative controls were HIV-1 sero-negative rhesus macaque sera and blank beads, or beads coupled to MulVg70 His6 (gp70 control). For detection of captured IgG or IgA antibodies, isotype-specific secondary antibodies were used. For analysis of IgG levels, titrations were made (5 times 6-fold starting from 1:80) and the binding magnitude is given as area-under-the-titration-curve (AUC) of the blank- and background-subtracted median fluorescence intensities (net MFI) of this titration curve. As reactivity towards conC was not measured for all time-points, it was excluded from magnitude-breadth plots, where the breadth is given as the proportion of the 9 readout antigens against which there is a response at the respective AUC value. Samples were classified as positive if the background-subtracted MFI and the net MFI were at least 3-fold over the week 0 sample background-subtracted MFI and net MFI and exceeded a cutoff established using seronegative samples. For analysis of IgA, only a 1:40 dilution of the plasma samples was tested because of the generally lower concentrations of this isotype, and the binding magnitude is given as net MFI. Rectal mucosal secretions were eluted from Weck-Cel sponges. Samples that exhibited blood contamination upon optical inspection were excluded from the analysis. For enhanced sensitivity of IgA detection, plasma and rectal weck elutions were depleted of IgG using protein-G sepharose columns. Levels of mucosal IgG antibodies are given as specific activity (SA) that is calculated as background-subtracted MFI measured for samples at a dilution of 1:5 divided by the total IgG concentration in µg/mL (as determined by IgG-ELISA). Samples were classified as positive if the MFI was ≥ 100 and SA was greater than or equal to an antigen-specific cutoff.

#### 2.4.5 Linear Peptide Epitope Mapping

The linear peptide epitope mapping was carried out by the CA-VIMC (Tomaras’s lab). The three top responding macaques from each group were selected based on the BAMA data. Serum collected at weeks 0 and 26 was added to a peptide microarray at a dilution of 1:250. Antibodies binding to the linear peptides were detected using a fluorescence-labelled anti-human-IgG antibody. The peptides on the array were 15-mers overlapping by 12 amino acids that covered consensus Env gp160 of clades A, B, C, D, group M, CRF1 and CRF2, as well as gp120 from strains 1.A244, 1.TH023, MN, 1086.C, TV1.C and 96ZM651.C. Results are given as the percentage of the highest fluorescence signal towards an epitope in a certain region of the sum of all fluorescence signals for each macaque.

#### 2.4.6 Neutralizing Antibodies

The capacity of serum samples to neutralize pseudoviruses was measured by the CA-VIMC (Montefiori’s lab) using the standardized TZM-bl assay as described previously (22, 23). Pseudoviruses produced in HEK293T cells carrying the following envelope proteins were assessed: MW965.26 (clade C, tier 1A), MN.3 (clade B, tier 1A), SHIV SF162.P4 (clade B, tier 1A), TH023.6 (clade E, tier 1B), BaL.26 (clade B, tier 1B), Ce1086_B2 (clade C, tier 2), TV1.21 (clade C, tier 2) and SHIV-SF162.P3 (clade B, tier 2). Dilutions of serum samples (from weeks 0, 6, 14, 26, 38 and 50) were added to the pseudoviruses before infection of TZM-bl cells. Luciferase activity was measured and the serum dilution at which relative luminescence units were reduced by 50% compared to virus control wells without serum sample was calculated and is given as inhibitory dilution 50 (ID_50_). In magnitude-breadth plots, the breadth is given as the proportion of the 3 (weeks 6 and 14) or 8 (weeks 0, 26, 38 and 50) pseudoviruses tested that are neutralized at the respective ID_50_.

#### 2.4.7 Antibody-dependent cellular cytotoxicity (ADCC) assay

The presence of antibodies capable of mediating ADCC was determined by the CA-VIMC ADCC core lab (Ferrari’s lab) using the GranToxiLux assay as described previously (24). Here, CEM.NKR cells were pulsed with recombinant gp120 matching the protein vaccine component, i.e., 1086 or TV1, and served as target cells. PBMCs were added as effector cells in the presence or absence of plasma samples. Thus, gp120-specific antibodies can recruit natural killer (NK) cells *via* their Fc-receptors to the target cells. To measure NK cell activation, the granzyme B activity was determined by adding a substrate that fluoresces upon cleavage. The net percentage of viable, fluorescent (i.e., granzyme B positive) target cells was measured by flow cytometry. The ADCC response is given as AUC of the granzyme B activity (% positive cells) curve over the log_10_-transformed plasma dilution series (5 steps 1:4 starting from 1:100). ADCC was analyzed for TV1 plasma obtained at weeks 0 and 26, and for 1086 additionally at weeks 36, 38, 48 and 50.

### 2.5 Statistical Analysis

The number of animals used represents the minimal number of animals required for sufficient statistical power (25). For the analysis of ELISpot and ICS data, the between-group differences in magnitude were determined using Tukey´s multiple comparisons test. For the rest of the assays, differences in response rates between groups were assessed using Fisher’s exact test, differences in magnitude between groups were determined using a non-parametric Wilcoxon rank sum test and differences before and after treatment within the same group were analyzed using the Wilcoxon signed-rank test in R. Correlations between mucosal and serum binding antibody measurements were assessed using Spearman’s rank correlation test. P-values were not adjusted for multiple comparisons.

## 3 Results

### 3.1 Study Design and Immunogens

Our previous HIV-1 vaccination studies in NHPs using DNA or NYVAC vectors for priming, followed by a booster with the same vectors in combination with an MF59-adjuvanted Env protein, demonstrated that this regimen readily induces potent T and B cell responses. However, the levels of HIV-1-specific humoral responses elicited after priming with DNA or NYVAC vectors alone were low and the induced B and T cell responses were considered not sufficiently durable (8, 10, 11).

With the primary goal to accelerate the induction of substantial levels of Env-specific antibodies and, at the same time, maintain the elicitation of balanced and potent T cell responses, we postulated that co-administration of Env protein with either a DNA or NYVAC vector already during the priming could result in the early generation of strong humoral responses. Therefore, the primary objective of this study was to identify the best prime-boost regimen in terms of frequency and magnitude of both humoral and cellular immune responses when combining the three vaccine components: (i) DNA, (ii) NYVAC and (iii) an MF59-adjuvanted Env protein.

For that purpose, 52 rhesus macaques were assigned to five groups consisting of 12 (groups 1 to 3) or 8 (groups 4 and 5) animals each. As depicted in **Figure 1**, all animals received two priming immunizations at weeks 0 and 4 with either NYVAC (N) or DNA (D) alone or in combination with MF59-adjuvanted Env protein (P). At weeks 12 and 24, groups were boosted with poxvirus NYVAC + protein (groups 1 to 4) or DNA + protein (group 5). Finally, re-immunizations were made at weeks 36 and 48 corresponding to the preceding ones for all groups, to assess whether responses can be maintained at high levels. For simplicity, we will refer to group 1 as G1-N2NP4 for the type of immunization performed; group 2 as G2-NP6; group 3 as G3-DP2NP4; group 4 as G4-D2NP4; and group 5 as G5-DP6 (**Figure 1**). All immunizations were performed by the intramuscular (i.m.) route and vaccine doses used are detailed in Materials and Methods. Peripheral blood mononuclear cells (PBMCs), mucosal and serum or plasma samples were obtained at selected time-points for the analysis of both cellular and humoral immune responses (**Figure 1**).

### 3.2 Safety Study

Safety of the vaccination regimen was not a primary endpoint of the study design; however, several parameters, including general systemic observations, local inoculation site reactions, physical examinations and clinical observations and pathology, were included as part of the animal health monitoring program. There were no significant changes in clinical pathology that would indicate an adverse effect related to the vaccines analyzed. Minimal dose site reactions were observed and any changes in clinical pathology were generally mild in nature, often pre-existing conditions or incidental findings. Overall, animals were devoid of any signs of clinical abnormality. The only scorable reporting was inappetence and occasional grade 1 local reactions. The days with the most inappetence correlate with anticipated recovery times from either fasting or sedation. Animal R129 from group 5 died at week 25 for causes non-related to the study. A more detailed description of the safety study results is included as **Supplementary Data 1**. In summary, no apparent toxicity was observed during the course of this study and the vaccines were well-tolerated as previously reported (11, 26, 27).

### 3.3 Magnitude and Quality of the HIV-1-Specific T Cell Responses by ELISpot Assay

Since an important requirement of vaccines against HIV-1 is their capacity to induce both HIV-1-specifc cellular and humoral responses in the vaccinees, both types of responses were thoroughly characterized. We first evaluated the magnitude and quality of the HIV-1-specifc T cell responses elicited in the five different NHP immunized groups by IFN-γ ELISpot assay. For this purpose, blood was collected at different time-points (**Figure 1**) and HIV-1-specific IFN-γ-producing cells in PBMCs along the immunization course were quantified.

**Figure 2:**
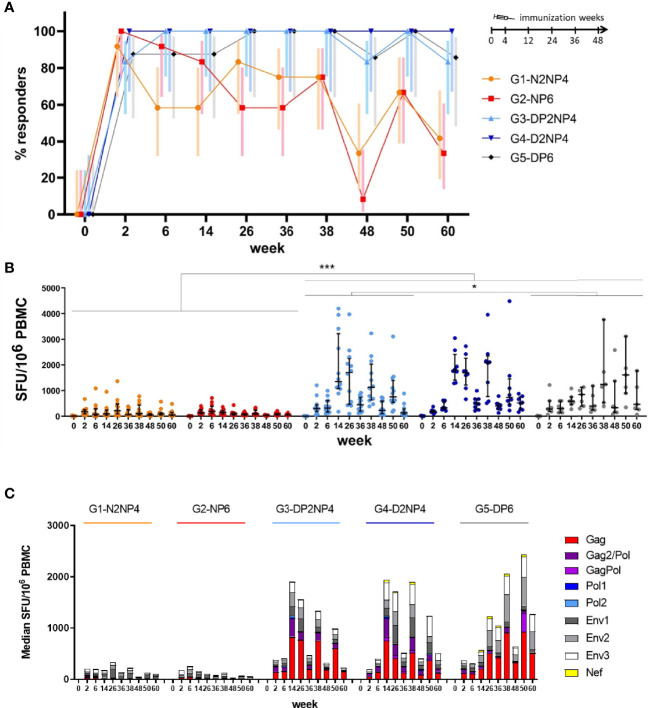
HIV-1-specific IFN-γ-producing cells along the study by ELISpot assay. **(A)** Percentage of responders by group and week with 95% confidence interval (CI). Criteria for responders were defined as 50 spot-forming units (SFUs) per million PBMCs and at least 4-fold increase above the baseline level for total HIV-1 response (sum of the nine different HIV-1 peptide pools). **(B)** Magnitude of the total HIV-1 response by group and week. PBMCs were freshly isolated at the indicated time-points, restimulated *ex vivo* with the nine different HIV-1 peptide pools and the number of HIV-1-specific IFN-γ-producing cells was quantified as SFUs/10^6^ PBMCs. Results are shown per animal (sum of the responses from the nine peptide pools) as colored dots, along with the median and interquartile range per group in black. Statistical significance was determined using Tukey´s multiple comparisons test. *, *p* < 0.05; ***, *p* < 0.001. **(C)** Magnitude of the total HIV-1 response by antigen, group and week. Absolute data from panel B are shown as median responses of each group towards the 9 peptide pools used.

DNA-primed groups showed higher percentage of responders (83%-100%) along the study than NYVAC-primed groups (8%-100%) (**Figure 2A**). With minor decreases in the percentage of responders at weeks 48 and 60 (i.e., 12 weeks post re-immunizations) the percentage of responders in the DNA-prime arm was stable from week 6 through the end of the study at week 60, while the percentage of responders in the NYVAC-prime arm waned with time and, consequently, the HIV-1-specific IFN-γ-producing cells also declined. Therefore, animals primed with DNA responded earlier than NYVAC-primed animals, and the response was maintained throughout the course of the study.

Comparative quantitative analysis of the IFN-γ-producing cells at all time-points revealed that groups 3, 4 and 5 (DNA-primed groups) exhibited higher levels than groups 1 and 2 (NYVAC-primed groups) (**Figure 2B**). Two weeks after completing the second DNA or NYVAC prime immunization (week 6), median responses of 206 (group 1), 256 (group 2), 406 (group 3), 401 (group 4) and 344 (group 5) SFUs per 10^6^ PBMCs were measured, indicating a more potent effect of the DNA prime over NYVAC prime (**Figure 2B**) as noted before (17). Co-administration of the protein had no apparent effect on the overall level of IFN-γ-secreting cells. The following 2 boosts at weeks 12 and 24 showed no remarkable effect in NYVAC-primed groups (groups 1 and 2), while in DNA-primed groups (groups 3, 4 and 5) boosting with NYVAC or DNA vectors + protein led to a vigorous increase (statistically significant), reinforcing the beneficial effect of DNA prime over NYVAC prime and being in line with previous studies in which the combination of different vector systems in heterologous prime-boost immunization regimens elicited more potent immune responses than homologous combinations (28). In particular, at week 14 (2 weeks post-1^st^ boost) median responses of 1,911 (group 3), 1,930 (group 4) and 612 (group 5) SFUs per 10^6^ PBMCs were measured, whereas groups 1 and 2 were below 200. Four animals across the groups exceeded 3,000 SFUs/10^6^ cells, with a peak of 4,195 SFUs/10^6^ cells (**Figure 2B**). Over longer time intervals (i.e., 12 weeks following the second boost and the re-immunizations), responses decline but can readily be boosted again to almost peak levels. Overall, there was a slow but steady decline in the responses from the peak after the 1^st^ NYVAC boost (week 14) in groups 3 and 4 until the end of the study. This could be due to the anti-vector immunity generated along the different NYVAC immunizations at weeks 12, 24, 36 and 48. In contrast, some animals of group 5 showed a steady increase in the responses from the peak after the 1^st^ DNA boost until the end of the study. By dissecting the responses from the nature of antigens, we observed that in NYVAC-primed groups (groups 1 and 2) most of the responses were directed against HIV-1 ENV antigen, while in DNA-primed groups (groups 3, 4 and 5) responses were distributed between ENV and GAG antigens (**Figure 2C**).

Overall, priming with DNA, with or without gp120 protein component, followed by boosters with NYVAC plus protein is the immunization regimen that triggers the most rapid and highest HIV-1-specific T cell responses. Re-immunizations help to maintain responses on high levels, but do not seem to lead to further improvements, except for some animals of group 5 which showed even further increases in magnitude with additional DNA plus protein immunizations. In general, however, vaccinations seem to be complete after three or four immunizations.

### 3.4 Magnitude and Quality of the HIV-1-Specific T Cell Responses by ICS Assay

Next, we analyzed the HIV-1-specifc T cell responses elicited by the different immunization regimens in terms of response rate, magnitude and balance between CD4^+^ and CD8^+^ T cells, peptide pool reactivity and polyfunctionality by ICS assay and flow cytometry. For this, cryopreserved PBMCs from selected time-points (weeks 6, 26, 36, 38, 48 and 50) were stimulated *ex vivo* with nine different peptide pools covering the HIV-1 antigens Gag, Pol, Env and Nef (1 Gag, 2 Gag/Pol, 2 Pol, 3 Env and 1 Nef peptide pools) and then stained for surface markers (CD3, CD4 and CD8) and intracellularly for the production of IFN-γ, IL-2 and TNF. As a negative control, cells were stimulated with DMSO (peptide pool buffer).

**Figure 3:**
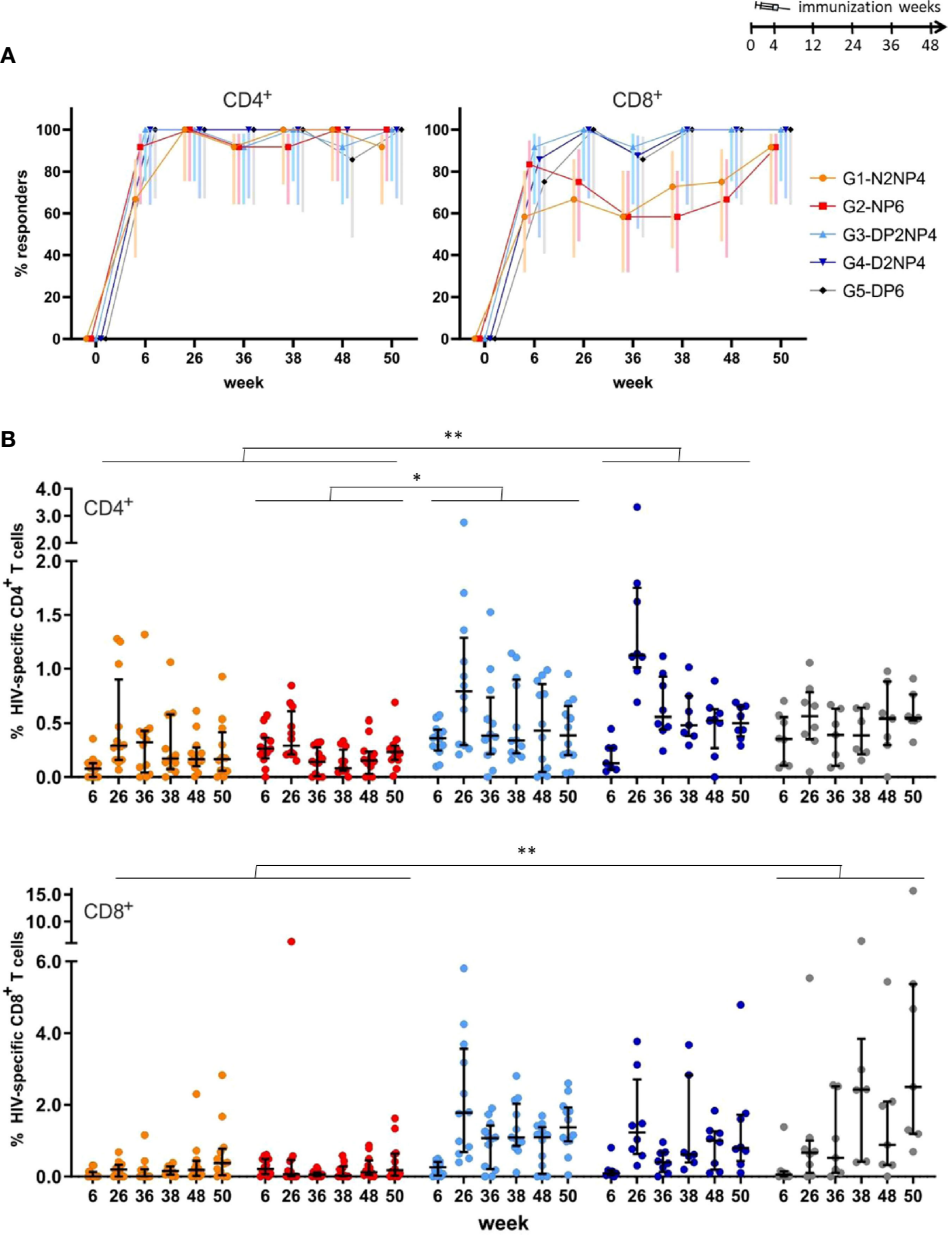
Overall HIV-1-specific CD4^+^ and CD8^+^ T cell responses by ICS assay. PBMCs obtained at the indicated time-points were stimulated *ex vivo* with the nine different HIV-1 peptide pools and stained for CD3, CD4 and CD8 and intracellular IFN-γ, IL-2, and TNF. Responding CD4^+^ and CD8^+^ T cells were quantified by flow cytometry. **(A)** Response rate (%) for all cytokines combined with 95% CI for CD4^+^ (left panel) or CD8^+^ (right panel) T cells. **(B)** Magnitude of the HIV-1-specific responses summed up for all the peptide pools and cytokines for CD4^+^ (upper panel) or CD8^+^ (lower panel) T cells per animal as colored dots with median and interquartile ranges in black. Statistical significance was determined using Tukey´s multiple comparisons test. *, *p* < 0.05; **, *p* < 0.005.

Comparing globally CD4^+^ and CD8^+^ T cell subsets along the study, the overall HIV-1-specific response rates for CD4^+^ T cells was 80%-100%, except for group 1 at week 6, where only 8 of 12 animals were classified as responders. For CD8^+^ T cells, a similar picture as in the ELISpot was observed, with groups 1 and 2 showing lower response rates (between 58% and 92%), though not significantly different (**Figure 3A**). Highest response rates for CD4^+^ T cells were observed towards ENV antigens, whereas for CD8^+^ T cells this was balanced mainly towards GAG and ENV (**Supplementary Figures 1, 2**, respectively).

Magnitudes of CD4^+^ and CD8^+^ T cells were similar for the NYVAC groups 1 and 2, whereas DNA-primed groups, especially group 3, showed a trend towards higher CD8^+^ than CD4^+^ T cell responses (**Figure 3B**). Both overall HIV-1-specific CD4^+^ and CD8^+^ T cell responses peaked at week 26 (2 weeks post-2^nd^ boost) in all groups except for the CD8^+^ T cell response observed in group 5 that reached the peak at weeks 38 (2 weeks post-1^st^ re-immunization) and 50 (2 weeks post-2^nd^ re-immunization), and responses were maintained in all groups at least for 50 weeks (**Figure 3B**). It is noticeable that some animals of group 5 with only DNA plus protein both in prime and boost reached the peak of CD8^+^ T cells at weeks 38 and 50 and maintained similar levels of T cell activation than groups 3 and 4 that incorporate NYVAC during boosters (**Figure 3B**). This delayed but maintained increase in the HIV-1-specific CD8^+^ T cell response elicited in some immunized animals from group 5 is consistent with the results of the ELISpot assay (**Figure 2B**). Regarding the different immunization protocols, again DNA-primed groups induced higher magnitudes of HIV-1-specfic CD4^+^ and CD8^+^ T cells than NYVAC-primed groups (statistically significant for group 4 vs groups 1 and 2, and group 3 vs group 2 for CD4^+^ T cells; and for group 5 vs groups 1 and 2 for CD8^+^ T cells).

**Figure 4:**
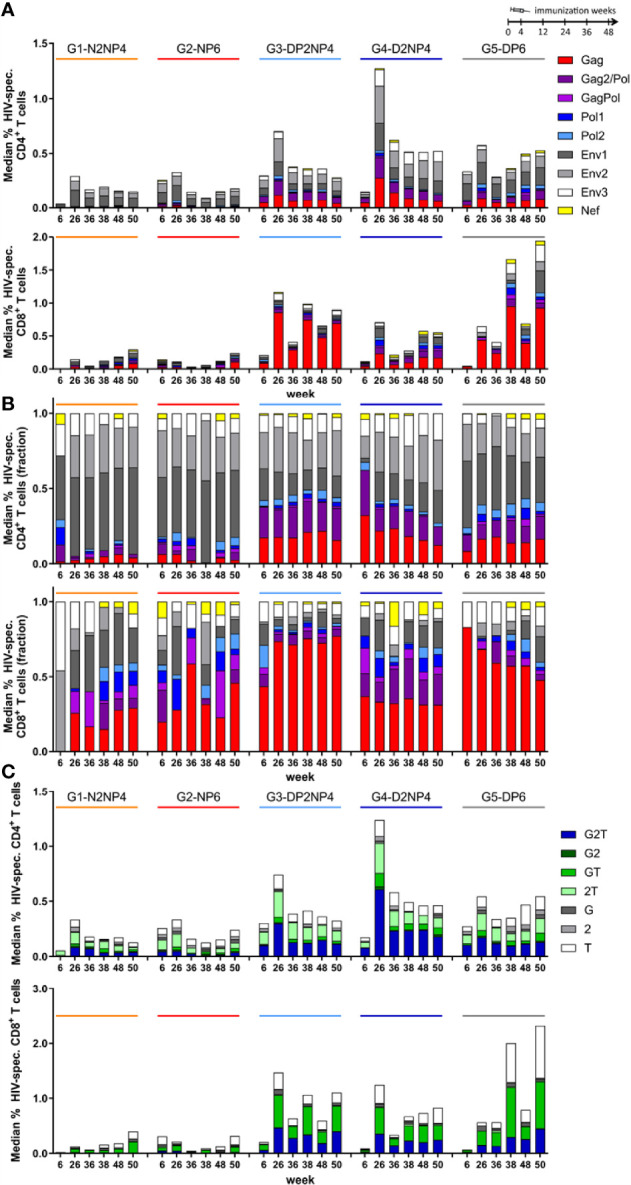
HIV-1-specific CD4^+^ and CD8^+^ T cell responses by ICS assay broken down according to peptide pool reactivity and cytokine profile. Absolute **(A)** or normalized **(B)** data from **Figure 3** shown as median responses towards the 9 peptide pools used (sum of all cytokines). **(C)** Absolute data from **Figure 3** shown as median responses of cells producing IFN-γ (“G”), IL-2 (“2”) or TNF (“T”) alone or in all combinations (sum of all peptide pools).

Analysis of the responses according to peptide pool reactivity showed that for CD4^+^ T cells, groups 1 and 2 were quantitatively dominated by ENV-specific responses, whereas the higher responses observed in the DNA-primed groups 3 to 5 were approximately one half directed towards ENV, and the other half towards GAGPOL. For CD8^+^ T cells, groups 1 and 2 had balanced responses against ENV and GAGPOL, while in groups 3 to 5 responses were mainly GAGPOL-mediated (**Figures 4A, B**). Overall, a significant proportion of animals did not develop POL- or NEF-specific responses.

The data were also analyzed regarding the number of cytokines produced by the restimulated T cells (**Figure 4C**, **Supplementary Figure 3**). On average over all time-points, the 30% of CD4^+^ T cells produced the three cytokines assessed (IFN-γ, IL-2 and TNF), whereas 39% produced a combination of two cytokines and 31% produced only one. Polyfunctionality of CD8^+^ T cells was similar, with 27% trifunctional, 43% bifunctional and 30% monofunctional cells.

In summary, and regarding overall differences between different prime/boost immunization regimens, we showed that: (i) NYVAC alone (group 1) showed similar response rates and magnitudes of response compared to NYVAC/gp120 prime (group 2); (ii) DNA alone (group 4) showed similar response rates and magnitudes of response compared to DNA/gp120 prime (group 3); (iii) DNA alone (group 4) showed statistically significant higher magnitudes of response than NYVAC alone (group 1); and (iv) DNA/gp120 prime (group 3) showed statistically significant higher magnitudes of response than NYVAC/gp120 prime (group 2). Above results indicate a potential benefit of the DNA prime (with or without protein) compared to the priming with NYVAC (with or without protein), confirming the results obtained by ELISpot analysis. Although the earlier administration of the MF59-adjuvanted Env protein component did not influence the magnitude and quality of T cell responses, a robust memory response is elicited as demonstrated by the rapid boosting effect observed after each re-immunization.

### 3.5 Magnitude and Quality of Antibody Responses

Vaccine-elicited protection against HIV-1 has been correlated with the presence of non-neutralizing antibodies (especially of subclass IgG3) targeting the V1/V2 region of Env as well as antibodies mediating ADCC (2, 20). Moreover, passive administration of neutralizing antibodies can protect against infection with sensitive virus strains (29). Thus, the characteristics of HIV-1-specific antibodies in the different macaque groups were evaluated at various time-points.

#### 3.5.1 Plasma/Serum Antibody Responses

Plasma samples for determination of IgG and IgA binding antibody levels were obtained at weeks 0, 6 (two weeks after the 2^nd^ prime immunization), 14 (two weeks after the 1^st^ vector+protein boost), 26 (two weeks after the 2^nd^ boost) and at weeks 36, 38, 48 and 50 (before and two weeks after the re-immunizations, respectively).

Total IgG levels were measured by BAMA against a panel of 10 readout antigens, including the gp120 proteins matching the protein vaccine component (i.e., 1086 and TV1 gp120), two V1/V2 antigens and a set of representative Env proteins of the other major clades.

**Figure 5:**
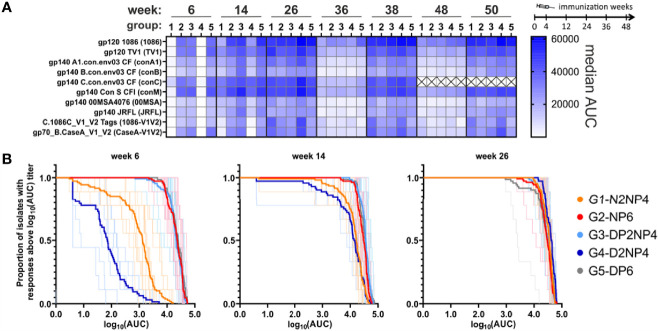
Binding antibody multiplex assay of IgG in plasma samples. **(A)** Magnitude of response to the various readout antigens (abbreviations used further are defined in brackets) measured as median AUC and indicated by color-intensity (scale on the right). ConC magnitudes were not determined for the late time-points (weeks 48 and 50; ×). **(B)** Magnitude-breadth plots of the overall IgG responses (log_10_-transformed AUC values of individual titration curves) towards the 9 readout antigens (excluding conC). Bold lines indicate overall group responses, while responses of individual monkeys are shown as thin lines.

Response rates were very high, with all animals being classified as responders with measurable IgG antibodies to at least one readout antigen already at week 6, except for one animal in group 4, where responses were generally lower. From week 14 onwards, seroconversion was confirmed for all animals with responses to almost all readout antigens (**Supplementary Figure 4**). Response magnitudes are shown in **Figure 5A** and summarized as magnitude-breadth curves in **Figure 5B**. IgG responses clearly lag behind in the groups without a protein component during the prime, with the DNA-only group 4 being inferior to the NYVAC-only group 1. All the other groups that received the 1086/TV1-gp120-protein immunization alongside the NYVAC or DNA primes already exhibit potent binding antibody responses at week 6. Two weeks after the 1^st^ boost immunization (week 14), when all groups received a protein injection in parallel to the respective vectors, groups 1 and 4 already come close to the other groups, and the difference disappears at week 26 (two weeks after the 2^nd^ boost immunization).

The same picture emerges when looking at individual readout antigens (**Figure 5A**, **6A**). Groups 1 and 4 lacking the protein priming component have statistically significant inferior responses at week 6, and for some readout antigens still at weeks 14 and 26. All groups exhibit similar kinetics. From week 26 onwards, responses decline over time, but rapidly increase again after additional immunizations (from week 36 before 1^st^ re-immunization to week 38 two weeks later, and from week 48 before 2^nd^ re-immunization to week 50 two weeks later). Interestingly, at these later time-points, responses only reach a level similar to the peak at week 26. Moreover, there is a trend of higher magnitude responses of group 4 from week 26 onwards for the Env antigens tested.

**Figure 6:**
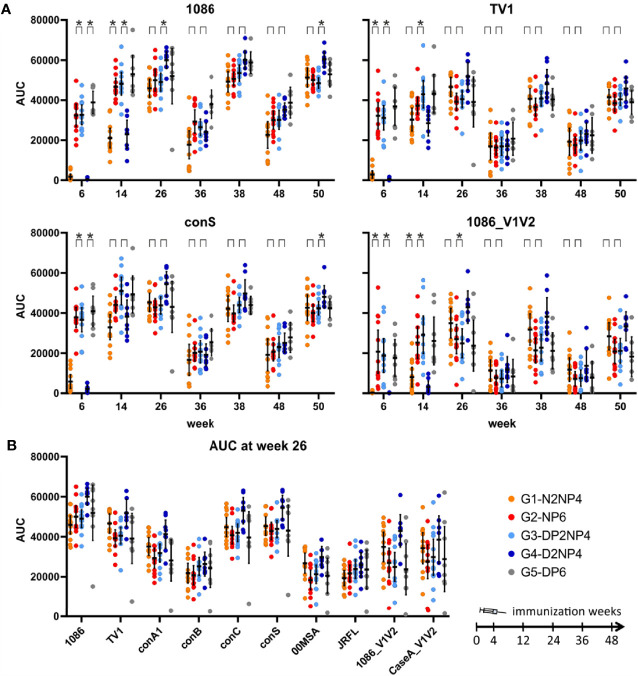
Binding antibody multiplex assay of IgG in plasma samples. **(A)** Time-courses for IgG responses (AUC values) towards selected readout antigens. Responses of individual monkeys are shown as colored dots and median and interquartile ranges are shown as black lines. Differences between groups 1 and 2, as well as between groups 3 and 4, were formally statistically tested; asterisks denote significant differences with *p*<0.05 (Wilcoxon rank sum test, Bonferroni-corrected for each antigen panel). **(B)** Comparison of week 26 responses (AUC values) towards the readout antigens.

A comparison of the magnitudes towards the various readout antigens (**Figure 5A**, **6B**) shows a similar pattern as previously observed (10, 11), with highest responses towards the homologous proteins used for immunization (1086 and TV1 gp120 as protein vaccine; clade C gp140(96ZM651) in the DNA and NYVAC vectors). V1/V2-directed antibodies are in the medium range of responses.

In conclusion, IgG antibody responses quickly reach high levels when the protein component is given in parallel to the DNA and NYVAC priming immunizations. However, the magnitude of humoral responses in groups 1 and 4 approach the levels of those of the other groups with the first boost that includes a protein component.

**Figure 7:**
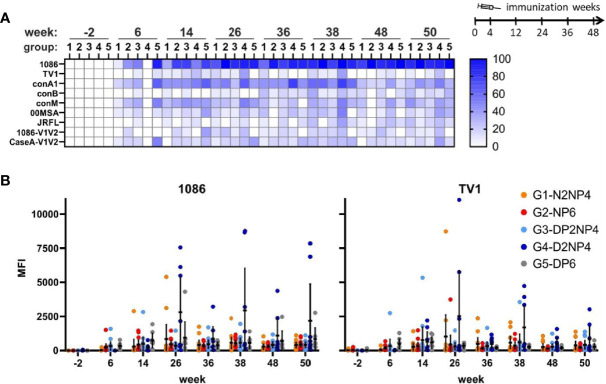
Binding antibody multiplex assay of IgA in plasma samples. **(A)** Response rates of animals to the various readout antigens are indicated by color-intensity (scale on the right). **(B)** Time-courses for IgA responses (mean fluorescence intensity [MFI] values of 1:40 diluted samples) towards selected readout antigens. Responses of individual monkeys are shown as colored dots and median and interquartile ranges are shown as black lines.

Serum IgA responses were also assessed from the same samples in parallel. Expectedly, IgA response rates were lower than for IgG (**Figure 7A**), with the highest response rate detectable towards the gp120(1086)-readout antigen, followed by conA1. As it is shown in **Figure 7B**, response magnitudes were, however, rather low, with average median values of the MFI (measured only at 1:40 dilution) at the peak time-points (weeks 26 and 38) in the range between 18 [CaseA-V1V2, week 26] to 541 [gp120(1086), week 38]. Interestingly, responses against the other component of the protein boost, gp120(TV1), were much lower, both regarding response rate and magnitude. Again, as above for IgG, there is a trend of a higher magnitude of IgA responses in group 4 from week 26 onwards for the two Env antigens tested (**Figure 7B**).

#### 3.5.2 Mucosal Antibody Responses

Mucosal antibodies could play an important role in the targeting and potential neutralization of HIV-1 before it enters the body. Thus, to get an impression of mucosal HIV-1-specific IgG and IgA levels, fluid from rectal mucosa was collected using Weck-Cel sponges (30) at weeks 6, 26, 38 and 50.

**Figure 8:**
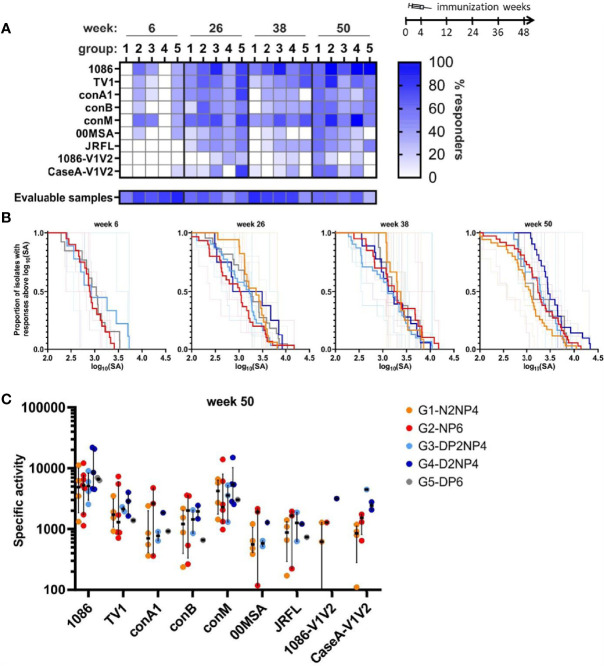
Binding antibody multiplex assay of IgG in fluid from rectal mucosa. **(A)** Response rates of animals to the various readout antigens are indicated by color-intensity (scale on the right). The proportion of animals for which evaluable samples were available is shown below the graph (same % scale). **(B)** Magnitude-breadth plots of the overall mucosal IgG responses (log_10_-transformed specific activity (SA) = MFI×dilution/[c(IgG)/µg/mL]) towards the 9 readout antigens. Bold lines indicate overall group responses, while responses of individual monkeys are shown as thin lines. Groups 1 and 2 were analyzed at week 6 but SA was too low. **(C)** Comparison of week 50 responses (SA) towards the readout antigens. Responses of individual monkeys for which evaluable samples were available are shown as colored dots and median and interquartile ranges are shown as black lines.

In 71% of the samples, HIV-1-specific IgG antibodies were detectable, with a mean concentration of 1.5 µg/mL as measured by ELISA. Some samples showed traces of blood contamination and were excluded from the analysis to avoid misleading results because of the presence of blood-derived antibodies. In total, 63% of samples were evaluable (**Figure 8A**). Response rates were low at week 6, with no responders detectable for groups 1 and 4 (**Figure 8A**). At the later time-points, on average 40% of the animals across groups had responses, with the highest fractions of animals responding to 1086, TV1 and conM, and the least responding to 1086-V1/V2. Specific binding of mucosal IgG antibodies towards the HIV-1 readout antigens was also measured by BAMA. The binding strength expressed as specific activity (SA) is the background-subtracted MFI normalized to the IgG-concentration in µg/mL. Magnitude-breadth plots show that the groups mostly behaved similar, except for, as mentioned for response rates, groups 1 and 4 at week 6. Group 4 had a trend to slightly higher responses at the final time-point (**Figure 8B**). The pattern of relative affinity towards the different readout antigens for the mucosal IgG (**Figure 8C**) was similar to serum IgG (**Figure 5B**). Binding antibody responses in mucosal and plasma samples are significantly positively correlated at each time-point with correlation coefficients ranging from 0.42 to 0.88 (data not shown).

Regarding mucosal IgA, 80% of the samples were evaluable and contained total IgA antibodies with a mean concentration of 4.0 µg/mL. However, no HIV-1-specific IgA could be detected in any sample.

#### 3.5.3 Epitope Mapping of Antibody Responses

**Figure 9:**
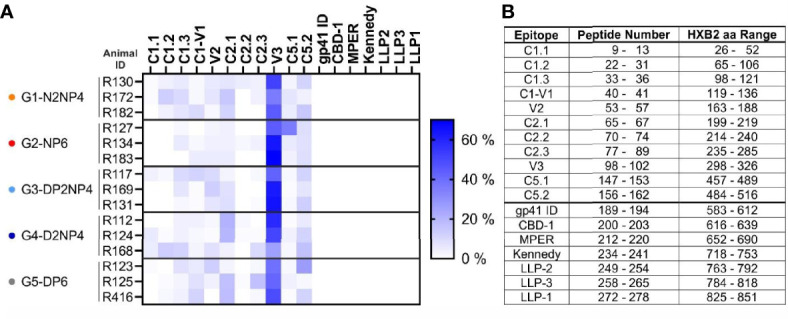
Linear epitope mapping. **(A)** Week 26 sera of the indicated macaques were analyzed for antibodies binding to linear Env epitopes on a peptide array. Week 0 responses were subtracted. Reactivity of IgG antibodies to subregions of Env is indicated by color intensity (scale on the right) for each animal analyzed. Binding to each subregion of Env is given in percent of the summed-up binding of each animal to all subregions. **(B)** Peptide numbers in the array library and aa range based on HXB2 numbering for each epitope.

To get an impression of the epitopes targeted by IgG antibodies, binding of serum samples to a linear epitope peptide array was analyzed. The array contains overlapping 15-mer peptides of a set of consensus Env gp160 sequences, as well as gp120 sequences of vaccine strains, including the clade C isolates 96ZM651, 1086 and TV1 that were used as vaccine antigens in this study. From each group, the three macaques with the strongest response in the BAMA assay were selected for further investigation and serum samples from weeks 0 and 26 were subjected to the analysis. As shown in **Figure 9**, multiple epitopes were targeted. The response was dominated by antibodies towards the V3 region, against which approximately half of the response was directed. Antibodies binding the remaining parts of gp120 were also detected, but at comparably lower levels, with C2.1 and C5.2 exhibiting yet the highest, and C1.1 and C2.2 the lowest responses. No responses against peptides derived from gp41 could be detected. There were no major differences of the binding pattern between the five groups.

#### 3.5.4 Neutralizing Antibody Responses

In addition to the analysis of antibodies binding to HIV-1 proteins, functional properties of the humoral responses were assessed.

**Figure 10:**
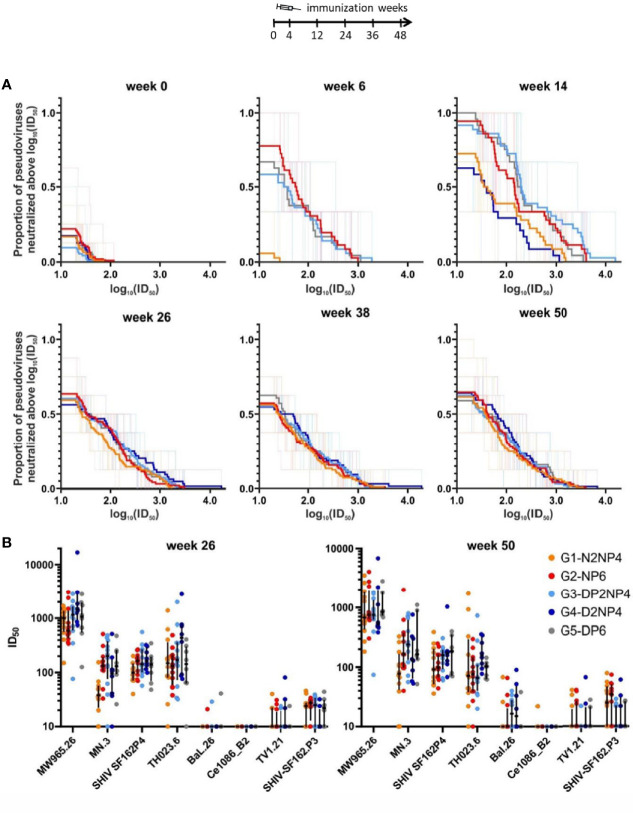
Neutralizing activity of serum samples. **(A)** Magnitude-breadth plots of log_10_(ID_50_) against the proportion of neutralized pseudoviruses in a TZM-bl assay. Eight pseudoviruses (MW965.26: clade C, tier 1A; MN.3: clade B, tier 1A; SHIV SF162P4: clade B, tier 1A; TH023.6: clade E, tier 1B; BaL.26: clade B, tier 1B; Ce1086_B2: clade C, tier 2; TV1.21: clade C, tier 2 and SHIV-SF162.P3: clade B, tier 2) were tested at weeks 0, 26, 38 and 50, whereas only three (MW965.26, SHIV-SF162P4 and TH023.6) were tested at weeks 6 and 14. **(B)** ID_50_ values of weeks 26 and 50 samples for neutralization of the different pseudoviruses. Responses of individual monkeys are shown as colored dots and median and interquartile ranges are shown as black lines.

First, the neutralization capacity of the serum samples from weeks 0, 6, 14, 26, 38 and 50 was tested in the TZM-bl assay, using a set of pseudoviruses carrying Env proteins from different clades that show different propensities to being neutralized, i.e., tier 1A, 1B and 2 viruses (31). The overall neutralization patterns are shown as magnitude-breadth plots in **Figure 10A**. Neutralization activity was absent for groups 1 and 4 at week 6, but developed until week 14, two weeks after the 1^st^ boost of a vector in parallel with the recombinant protein. Although the responses of both groups were slightly inferior at week 14, there were no differences between the five groups at the later time-points. The pattern did not change from week 26 to weeks 38 and 50, thus the re-immunizations did not affect the quality or quantity of the neutralization responses. Regarding the neutralization activity towards the different pseudoviruses (**Figure 10B**), the clade-matched, easy-to-neutralize Env isolate MW965.26 was potently neutralized. Viruses pseudotyped with the MN.3, SHIV SF162P4 and TH023.6 envelopes were also neutralized, with median 50% inhibitory dilutions mostly above 1:100. Moderate neutralization activity was found against the other strains in some monkeys except for Ce1086_B2.

#### 3.5.5 Antibody-Dependent Cellular Cytotoxicity

**Figure 11:**
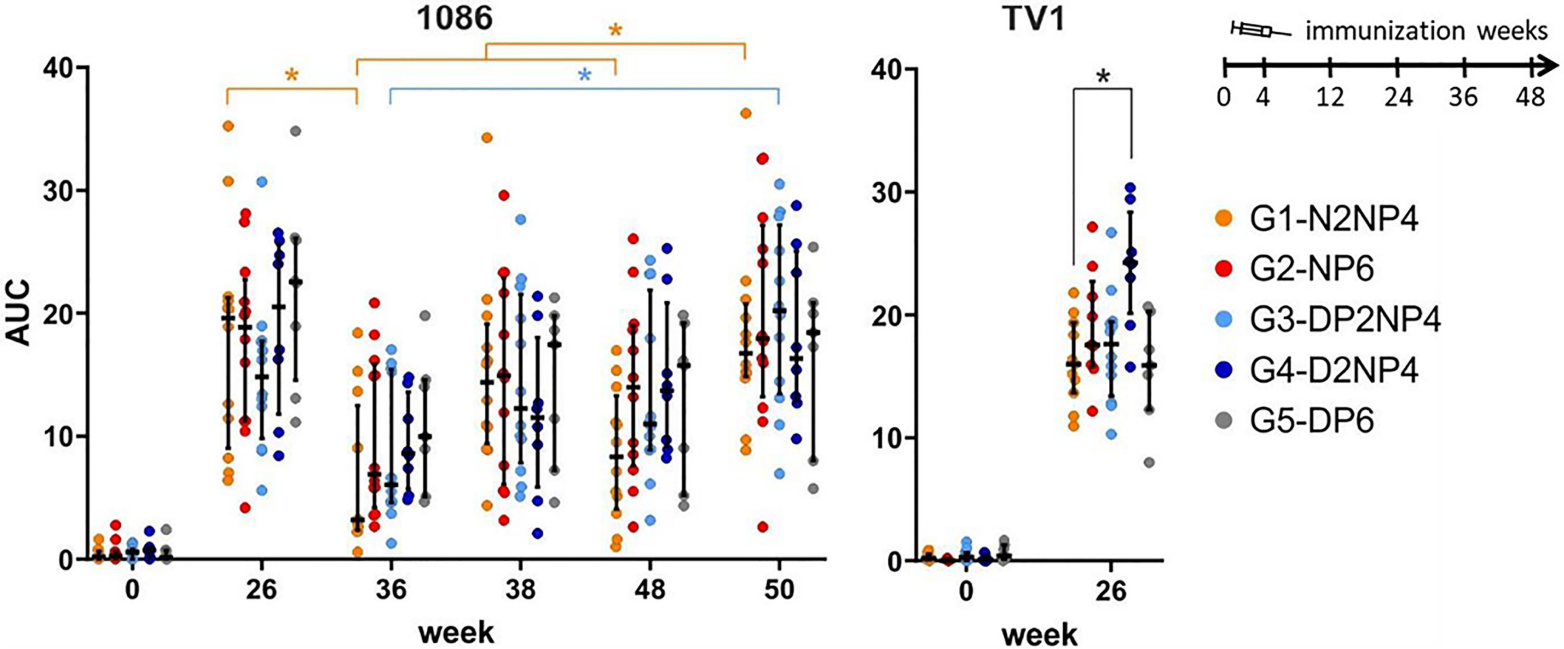
Capacity of plasma samples to mediate ADCC. AUC values of the percent granzyme-B-positive target cells plotted against plasma dilution in the ADCC GranToxiLux assay, using gp120 of 1086 (left) and TV1 (right) as readout antigens coated onto target cells. Responses of individual monkeys are shown as colored dots and median and interquartile ranges are represented as black lines. The within-group comparisons (left panel) were made using the Wilcoxon signed-rank test and the between-group comparison (right panel) was performed using the Wilcoxon rank sum test (Bonferroni-corrected). *p<0.05.

Next, the ability of plasma samples to mediate antibody-dependent cellular cytotoxicity (ADCC) was analyzed at weeks 0, 26, 36, 38, 48 and 50 by employing the ADCC GranToxiLux (GTL) assay (24). Target cells pulsed with gp120(1086) or gp120(TV1) were co-cultured with effector cells in the presence of the monkeys’ plasma. All groups clearly showed ADCC activity at week 26 towards both readout antigens (**Figure 11**). Interestingly, for TV1, group 4 exhibited higher ADCC activity than the other groups (statistically significant against group 1). Durability of the ADCC responses was assessed using 1086 as target. As observed for binding antibodies, ADCC-functional antibody levels declined by trend from week 26 to week 36 (significant for group 1) but could readily be boosted again. Similarly, the final re-immunization led to some increase in ADCC levels, with a significant increase in group 1 as compared to weeks 36 and 48, and in group 3 as compared to week 36. There were no statistically significant differences between the groups at any time-point.

## 4 Discussion

It is generally agreed that an HIV vaccine should trigger both arms of the immune system, humoral with induction of broadly neutralizing antibodies (bNAbs) and cellular with activation of specific CD4^+^ helper and CD8^+^ cytotoxic T cell responses. While many vaccine candidates have been shown to induce both B and T cell responses to HIV, these immune responses wane with time and, thus far, no efficient HIV vaccination protocol has been developed that prevents human HIV infection since the results of the RV144 phase III clinical trial first suggested that this might be achievable.

We, as part of the Collaboration for AIDS Vaccine Discovery (CAVD), have been evaluating different nucleic acid vaccines, non-replicating viral vectors and Env protein components in mixed modality vaccine regimens to define best-in-class strategies to accelerate and potentiate HIV-1-specific immune responses that could help in the control of HIV infection. In previous NHP studies that employed the same DNA and NYVAC vaccine vectors expressing the same antigens as described in this report, it was observed that priming with the DNA vaccine qualitatively framed the immune response, especially inducing potent and polyfunctional T cell responses well-balanced against the different antigens (10, 11). Humoral responses, however, only reached peak levels late during the study after the administration of adjuvanted protein after NYVAC boost injection (in that case only TV1 gp120 in MF59) (10) or administered in parallel with NYVAC vectors (in that case both TV1 and 1086 gp120 in MF59) (11). As humoral responses are considered major contributors to a complex overall immune response capable of protecting from HIV (32, 33), the main question of the present study was to assess whether it is possible to combine the best of both arms of the immune system by co-administering the DNA or NYVAC vaccine with the MF59-adjuvanted Env protein already during the priming immunizations. With this aim, we have evaluated both cellular (response rate, magnitude, breadth and quality of CD4 and CD8 T cell responses) and humoral (IgG and IgA binding antibodies, neutralization activity and ADCC function) immune responses elicited by different DNA/NYVAC/Protein immunization regimens in rhesus macaques in order to determine the potential benefit of including the protein component at priming to achieve early immune responses. In addition, we have also evaluated the benefit of late boosters for the maintenance of the immune responses against HIV.

As it was previously observed that co-administration of recombinant gp120 of the C-clade isolates 1086 and TV1 led to broader humoral immune responses (11), this mixture was used again in the present study. Of course, recombinant next-generation trimeric gp140 protein that is stabilized in the closed, pre-fusion conformation and that exposes vulnerable sites for the elicitation of bNAbs should be a preferable antigen (34). However, such stabilized proteins were not available from C-clade isolates at the time of the study since they have been developed later (35).

The accompanying monkey health monitoring demonstrated that the vaccines were well-tolerated, and no apparent toxicity was observed during the study. Minimal dose site reactions were reported and changes in clinical pathology were generally mild in nature.

Regarding overall HIV-1-specific T cell responses, we observed higher response rates and magnitudes in DNA-primed groups (groups 3, 4 and 5) than in NYVAC-primed groups (groups 1 and 2) by both ELISpot and ICS assays. The observation that a vaccination regimen including a DNA prime followed by a NYVAC boost considerably enhanced vaccine-elicited T cell immune responses compared to an immunization protocol using NYVAC alone has been previously reported both in NHP studies (8, 10) and in human clinical trials (36–38).

By ELISpot assay, DNA-primed groups showed higher percentages of responders (83%-100%) than NYVAC-primed groups (8%-100%) along the study, and the percentage of responders in the DNA-prime arm was stable from week 6 through the end of the study (60 weeks), whereas in NYVAC-primed groups this percentage decreased with time. This positive effect of the DNA prime over NYVAC prime is also observed when we analyzed the magnitude of the total HIV-1-specific response. Comparative quantitative analysis of the IFN-γ-producing cells revealed that DNA-primed groups induced statistically significant higher levels than NYVAC-primed groups. Two weeks after the second DNA or NYVAC prime immunization, median responses of SFUs per 10^6^ PBMCs in NYVAC-primed groups (231) were lower than those in DNA-primed groups (384), indicating a more potent effect of the DNA prime over NYVAC prime. The following 2 boosts and the late re-immunizations had no remarkable effect in NYVAC-primed groups, while in DNA-primed groups, boosting with NYVAC or DNA vectors + protein induced a statistically significant potent increase, supporting the beneficial effect of the DNA prime over NYVAC prime. The observation that NYVAC + protein immunization clearly boosted DNA-primed immune responses has been previously reported in an NHP study using the same HIV-1 antigens in a heterologous DNA prime followed by NYVAC + protein boost immunization regimen but without the Env protein component being administered at priming (11). The lowest response obtained in group 5 (six immunizations with DNA + protein) at week 14 (2 weeks post-1^st^ boost), compared to groups 3 and 4, is consistent with the homologous nature of the immunization regimen. However, over the following three vaccinations, median responses declined with time in groups 3 and 4, while group 5 showed a clear increase of the response with time, reaching the maximum level at week 50. This could be due to the anti-vector immunity against NYVAC vector generated along the different immunizations in groups 3 and 4. Therefore, the protein co-administration during priming had no effect on HIV-1-specific T cell responses. By dissecting the responses from the nature of antigens, we observed that most of these responses were directed against HIV-1 antigen ENV in NYVAC-primed groups, or against ENV and GAG antigens in DNA-primed groups. This immunodominance of ENV over GAG responses in NYVAC-primed groups could be related to the design of the vaccine candidates. NYVAC-gp140(96ZM651) and NYVAC-Gag(96ZM651)-Pol-Nef(97CN54) viruses (15) expressed a soluble trimeric gp140 protein or a Gag-Pol-Nef protein that is processed into the 55-kDa Gag protein that is able to induce VLPs, respectively. Although both viruses are administered in equal concentrations (a mix of 1×10^8^ pfu of each virus), the number of gp140 molecules that are expressed and sensed by the immune system in a specific window of time is higher than the number of Gag-Pol-Nef molecules produced, since Gag-Pol-Nef gene is larger than gp140 gene. This fact could be responsible, at least in part, for the immunodominance of ENV observed in NYVAC-primed group. This effect could be counteracted by adjusting the molar ratio of the vaccine candidates and/or by temporal or spatial separation of the Env and Gag antigens.

When we analyzed the HIV-1-specific T cell responses by ICS and flow cytometry, we observed well-balanced CD4 and CD8 T cell responses, as previously reported in other NHP studies using similar immunogens and immunization regimens (11). The overall HIV-1-specific response rates were slightly higher for CD4^+^ T cells compared to CD8^+^ T cells, especially in NYVAC-primed groups. However, the magnitude of the response was slightly higher for CD8^+^ T cells compared to CD4^+^ T cells, especially in the DNA-primed groups. Total HIV-1-specific CD4^+^ and CD8^+^ T cells peaked 2 weeks post-2^nd^ boost in all groups except for the CD8^+^ T cell response observed in group 5 that reached the peak 2 weeks post the late re-immunizations, confirming the delayed but maintained response elicited by this homologous immunization regimen previously observed in the ELISpot assay. Again, protein co-administration at priming had no effect on HIV-1-specific T cell responses, as differences were only observed between DNA-primed vs. NYVAC-primed groups (statistically significant for group 4 vs groups 1 and 2, and group 3 vs group 2 for CD4 T cells; and for group 5 vs groups 1 and 2 for CD8 T cells). In terms of antigens, for CD4^+^ T cells groups 1 and 2 (NYVAC-primed groups) were quantitatively dominated by ENV-specific responses, whereas the higher responses observed in the DNA-primed groups (groups 3 to 5) were evenly distributed between ENV and GAGPOL. Again, ENV immunodominance in NYVAC-primed groups is observed. For CD8^+^ T cells, groups 1 and 2 had balanced responses against ENV and GAGPOL, while in groups 3 to 5 responses were mainly GAGPOL-mediated.

HIV-1-specific T cell responses were polyfunctional with approximately a third of T cells producing all three cytokines assessed (IFN-γ + IL-2 + TNF), combinations of two cytokines, or only one, respectively. This polyfunctional phenotype has been associated with protective antiviral responses (39).

Therefore, analysis of T cell responses indicates a potential benefit of the DNA prime (with or without protein) compared to the priming with NYVAC (with or without protein). Overall, priming with DNA, in the presence or absence of the Env component, followed by boosters with NYVAC + protein is the immunization regimen that triggers the most rapid and highest HIV-1-specific T cell responses. The earlier administration of the MF59-adjuvanted Env protein does not influence the magnitude and quality of T cell responses, but a robust memory response is induced as shown by the boosting effect after each re-immunization.

For many vaccines, levels of binding antibodies constitute a correlate of protection (33). Yet, in the case of HIV, the correlate, as far as it has been deduced from RV144 trial, is a complex network of certain components from the humoral and cellular immune responses that together contribute to protection (2, 3, 20, 40–42). Moreover, it is so far not clear whether the observed correlates of reduced risk are mechanistic or rather correlating with other effectors. Nevertheless, the overall levels of binding antibodies constitute a dynamic range, within which functional responses have their place and may contribute to protection in this complex and interdependent pattern. In addition, previous studies by others in mice and macaques have reported robust and early antibody development if a protein component was co-administered with DNA during priming compared to the administration of either component alone or with a DNA prime/protein boost regimen (43–45). It has also been reported in a recent phase 1b clinical trial that co-administration of gp120 Env protein component with DNA or NYVAC vectors during priming led to early and potent induction of Env V1/V2 IgG binding antibody responses (38). However, in our NHP study most of the antibody responses were directed against the V3 region of gp120 while levels of antibodies to the V1/V2 region were lower than observed in the human trial.

Looking at the kinetics of the overall antibody responses, groups 2, 3 and 5 that received the protein component during the first two immunizations, already exhibited high antibody levels at week 6. These responses increased only marginally (magnitude/breadth-AUCs by 4% on average) peaking at week 14. In contrast, groups 1 and 4 that received the 1^st^ protein immunization at week 12, lacked behind and reached their peak levels at week 26 (two weeks after the 2^nd^ protein injection). This justifies co-administration of the MF59-adjuvanted protein already during the first DNA priming immunization, as previously reported (38, 43–45), to elicit potent antibody responses as early as possible. From their respective peaks onwards, responses declined over time but could readily be increased again to their peak levels with additional re-immunizations. Thus, these responses seem to be anamnestic in nature, indicating that a rapid increase in humoral responses might also occur upon infection. Moreover, this shows that additional boosts do not lead to a further increase in response magnitude – at least not for overall binding IgG levels – but can contribute to keeping the antibody levels at high values over time through the induction of a potent memory response. This could be important given that there is only a short window of opportunity to prevent infection before HIV establishes itself (46). In line with our previous studies (8, 10, 11), reactivity of sera was generally broad, highest towards the homologous readout antigens, followed by C-clade antigens and, interestingly, the M group consensus, whereas the reactivities were inferior to heterologous clades B and A readout antigens.

Antibody target sites were assessed using a linear epitope peptide microarray. It is important to keep in mind that antibodies recognizing complex structural epitopes, as it is often the case for bNAbs, may likely remain undetected in this assay. The highest signals were detected for peptides from the V3 region that theoretically constitutes a vulnerable site, though bNAbs targeting this region usually also recognize glycans (47) that are not represented on the microarray. Most other gp120 regions, including V1 and V2, were also targeted, although at lower levels. In contrast, no responses were observed against gp41-derived peptides, most probably because this part was lacking from the protein boost component that is the main driver of humoral responses.

Among the RV144 vaccinees, high serum IgA antibodies constituted a correlate of risk, as these antibodies competed with otherwise protective ADCC-mediating antibodies (20, 48). Serum IgA levels elicited by the vaccines in the present study were quite low compared to IgG levels. However, mucosal IgA antibodies that could conceptually be considered important contributors to block HIV at the sites of entry could not be detected at all. Thus, development of mucosal vaccine delivery strategies could be important to add this type of humoral responses to the overall profile.

To complement the characterization of the humoral immune reactions, two important functional antibody responses, neutralization and ADCC, were assessed.

As observed for binding antibodies in general, serum neutralization only developed after administration of MF59-adjuvanted Env protein, so groups 1 and 4 initially lacked behind but catch up at the later time-points. Neutralization was potent against most tier 1 isolates, but tier 2 virus neutralization levels were low in few macaques. The overall observed neutralization pattern is consistent with data obtained using the same vaccine components in another regimen (11), except that the magnitude of the neutralization response is slightly higher in the present study and develops quicker, presumably because of the MF59-adjuvanted Env protein being administered much earlier.

Consistent ADCC responses could be observed in all groups in the GranToxiLux assay using target cells pulsed with the homologous vaccine antigens. For readout against 1086 gp120, all groups behaved similarly. Interestingly, however, group 4 (DNA prime without protein) showed a significantly higher ADCC response against the TV1 gp120-coated target cells. Remarkably, a similar phenomenon was observed in the clinical phase 1b trial HVTN096, where people receiving a DNA prime without protein versus those who received protein alongside the DNA had a higher ADCC response magnitude against two of three readout antigens as well (38).

The efficacy of our immunization regimens and vaccine candidates was not assessed because the objective of the study was to validate the different vaccine candidates and extend the knowledge on the optimal immunization regimen for further studies and because of the limited conclusions that we would have obtained for the HIV-1-derived antigens after a challenge with a SHIV strain. Nevertheless, results of this study influenced the design of the phase IIB PrEPVacc-trial (Clinical trials identifier: NCT04066881), which includes an arm with DNA+protein for all 4 immunizations (DNA identical to this study), and another one with DNA+protein twice, followed by MVA+protein twice, so in principle matching groups 3 and 5.

The strenght of this AUP512 study is the comparative head-to-head analysis of different vaccination regimens and their long-term B and T cell immune behavior after repeated booster immunizations. In this sense, the information provided in this investigation has also implications for the consideration of repeated booster doses when vaccines are administered and cannot confer long-term immunity. In the case of SARS-CoV-2/COVID-19 pandemic, it was shown that booster doses based on homologous mRNA or adenovirus vaccines were required to maintain sufficient levels of antibodies to control the coronavirus infection. Our studies here with vectored HIV vaccines showed that there is no benefit of re-immunizations (weeks 36 and 48) in terms of further increasing the magnitude or shaping the quality of the response (three to four immunizations constituted a complete immunization regimen). However, re-immunizations recall the magnitudes to peak levels, showing the presence of robust memory. This may also be applicable to coronavirus vaccines. If we had a better understanding on correlates of protection and thereshold values above which those correlates need to be, re-immunizaton/refreshing intervals could be rationally devised.

In conclusion, early administration of the MF59-adjuvanted Env protein in parallel with the DNA primes leads to a quicker buildup of the humoral immune responses, without negatively affecting the cellular responses. Thus, future clinical trials employing these or similar vaccine components, including replication-competent poxvirus-based vectors (8), should be designed following the group 3 regimen: DNA+protein prime, followed by up to two poxvirus+protein boost immunizations. Moreover, late boosters help to maintain B and T cell immune responses. Thus, our findings support the use of the Env protein component during priming in the context of an heterologous immunization regimen with a nucleic acid or viral vector as an optimized immunization protocol against HIV infection.

## Data availability statement

The original contributions presented in the study are included in the article/**Supplementary Material**. Further inquiries can be directed to the corresponding authors.

## Ethics Statement

The animal study was reviewed and approved by ABL, Inc.’s Institutional Animal Care and Use Committee (Rockville, MD, USA). All procedures and primate colony care strictly adhered to the recommendations found in the 8th edition of the Guide for the Care and Use of Laboratory Animals (National Research Council), as well as in the Public Health Services Policy on the Humane Care and Use of Laboratory Animals from the Office of Animal Welfare (part of the USA Department of Health and Human Services), with all activities being in full compliance with the regulations found in the Animal Welfare Act (9 CFR 3.81).

## Author Contributions

Conceptualization: GP, JH, SD, ME, RW, MK, SB, AC, RG. Formal analysis: BP, BA, CG, JK, DW, KF, MR, DM, NY, GF, XS, SS, GT, AS. Funding acquisition: GP, MR, DM, GT, SB. Investigation: BP, BA, CG, JK, DW, AC, KF, MR, DM, NY, GF, XS, SS, GT, AS. Methodology: BP, BA, CG, JK, DW, KF, MR, DM, NY, GF, XS, SS, GT, WF. Resources: GP, MR, DM, GT. Supervision: GP, JH, ME, RW, GT. Validation: AS, GP, JH, ME, RW, DM, GF, GT. Writing—original draft: BP, BA, ME and RW. Writing—review and editing: all authors. All authors contributed to the article and approved the submitted version.

## Funding

This investigation was funded by the Bill & Melinda Gates Foundation Poxvirus T Cell Vaccine Discovery Consortium (PTVDC) (38599). The Vaccine Immune Monitoring Centers (OPP1032144 and OPP1032325) and the Vaccine Immunology Statistical Center (OPP1032317), as part of the Collaboration for AIDS Vaccine Discovery (CAVD), were funded by the Bill & Melinda Gates Foundation. We also acknowledge support from NIH/NIAID Duke CFAR P30 AI064518. Novartis Vaccines and GSK (subsequently to acquisition of Novartis Vaccines) received support for this work under contract number HHSN266200500007C from DAIDS-NIAID-NIH to develop and prepare the gp120 antigen material.

## Author Disclaimer

The funders had no role in study design, data collection and interpretation or the decision to submit the work for publication.

## Conflict of Interest

Author SB was employed by company Novartis Vaccines. Author DW and AC were employed by company ABL Inc. MK is an employee of the GSK group of companies and holds shares in GSK.

The remaining authors declare that the research was conducted in the absence of any commercial or financial relationships that could be construed as a potential conflict of interest.

## Publisher’s Note

All claims expressed in this article are solely those of the authors and do not necessarily represent those of their affiliated organizations, or those of the publisher, the editors and the reviewers. Any product that may be evaluated in this article, or claim that may be made by its manufacturer, is not guaranteed or endorsed by the publisher.
