# Supplementary material for: Early and Long-Term HIV-1 Immunogenicity Induced in Macaques by the Combined Administration of DNA, NYVAC and Env Protein-Based Vaccine Candidates: The AUP512 Study
Source: Front Immunol. 2022 Jul 22;13:939627. doi: 10.3389/fimmu.2022.939627 (PMC9354927; doi:10.3389/fimmu.2022.939627)
Supplement: Supplementary file 1 [file DataSheet_1.docx]

***Supplementary Material***

# Supplementary Data

**SUPPLEMENTARY DATA 1. Animal health monitoring program**

General systemic reactions consisted mostly of intermittent inappetence, which was mostly related to sedation or increased activity in the animal room. Local inoculation site reactions consisted mainly of mild erythema at the site of inoculation, usually within the first 2 days following inoculation. No score higher than a grade 1 was assigned to any local reaction throughout the duration of this study. There were no significant findings based on the physical examinations. Several monkeys were considered underweight and were given food supplementation. Occasional dermal rashes were also observed which are not unusual in monkeys and were not considered to be related to the vaccines tested or their administration. Incidental changes in several parameters, including fasting hemoglobin, urea nitrogen, glucose and sodium levels, were detected being distributed across groups and not associated with a specific treatment modality, as they were observed both pre- and post-immunization. The clinical pathology results also revealed several macaques across different groups that showed elevated background fibrinogen levels in the pre-immunization collection time-point. Therefore, it was not surprising that several of these same macaques also experienced elevated fibrinogen levels post-immunization. Many of the macaques with elevated fibrinogen levels did not have corresponding elevated C-Reactive Protein (CRP) levels at the same time-points post-immunization. Elevated fibrinogen and CRP levels were sporadic and did not correlate/associate with specific immunizations or vaccine groups. Out of range results in these clinical parameters were transient and resolved over time. A few macaques had elevated or decreased leukocyte counts in the pre-immunization collection time-point that were scored as grade 1 changes. All but one of these macaques did not demonstrate continued elevation or decline in leukocyte counts post-immunization. One of the macaques demonstrated another grade 1 increase in leukocytes at a separate time-point but then returned to normal levels by the next evaluation point. Elevated leukocyte levels were sporadic and did not correlate/associate with specific immunizations or vaccine groups. One animal in group 1 showed continued elevation of leukocytes post-immunization with NYVAC + protein regimen, but this was only noted up to 1 week post-2^nd^ immunization. Levels returned to a normal range by 1 week post-3^rd^ immunization and remained within the normal range for the remainder of the study.

# Supplementary Figures

**
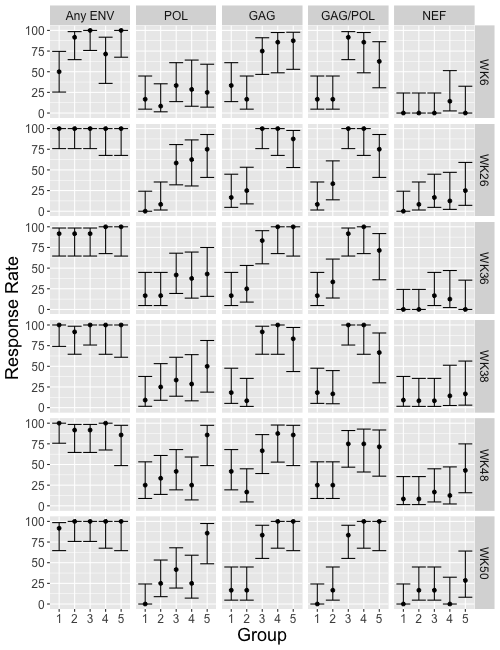
**

**SUPPLEMENTARY FIGURE 1. Response rate (%) of combined cytokine analysis against different HIV-1 peptide pools, with 95% CI by group, week and stimulation for CD4^+^ T cells.**

**
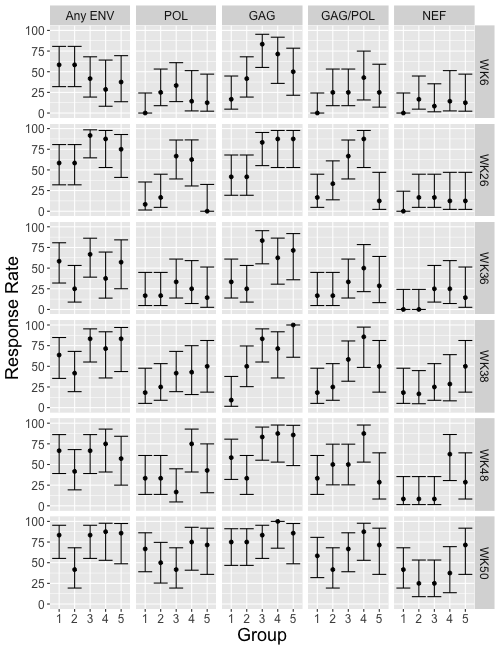
**

**SUPPLEMENTARY FIGURE 2. Response rate (%) of combined cytokine analysis against different HIV-1 peptide pools, with 95% CI by group, week and stimulation for CD8^+^ T cells.**

**SUPPLEMENTARY FIGURE 3. HIV-1-specific CD4^+^ (upper panel) and CD8^+^ (lower panel) T cell responses by ICS assay broken down according to cytokine profile.** Normalized data are represented as median responses of cells producing IFN-γ (“G”), IL-2 (“2”) or TNF (“T”) alone or in all combinations (sum of all peptide pools).

**SUPPLEMENTARY FIGURE 4. Binding antibody multiplex assay of IgG in plasma samples**. Response rates of animals to the various readout antigens are indicated by color-intensity (scale on the right). ConC responses were not measured for the late time-points (weeks 48 and 50; ×).
